# Transdermal Delivery of Hyaluronate-Conjugated Formyl Peptide Receptor 2 Agonistic Peptide Ameliorates Bleomycin-Induced Skin Fibrosis

**DOI:** 10.34133/bmr.0316

**Published:** 2026-03-12

**Authors:** Gyu Tae Park, Hye Eun Choi, Jae-Kyung Lim, Eun-Bae Choi, Jeong-Hyun Park, Su Bin Lee, Moon-Bum Kim, Ki Su Kim, Jae Ho Kim

**Affiliations:** ^1^ Department of Physiology, College of Medicine, Pusan National University, Yangsan 50612, Gyeongsangnam-do, Republic of Korea.; ^2^ School of Chemical Engineering, College of Engineering, Pusan National University, Busan 46241, Republic of Korea.; ^3^ Department of Dermatology, Pusan National University Hospital, Busan 49241, Republic of Korea.; ^4^ Convergence Stem Cell Research Center, Pusan National University, Yangsan 50612, Gyeongsangnam-do, Republic of Korea.

## Abstract

Systemic sclerosis is a severe autoimmune disorder defined by progressive tissue hardening and inflammatory infiltration that affects the skin and major internal organs. The formyl peptide receptor 2 (FPR2) agonist, WKYMVm (Wm), shows promise for alleviating dermal fibrosis; however, its clinical translation is impeded by the need for invasive subcutaneous injection to bypass the skin barrier. To overcome this limitation, we developed a noninvasive topical delivery system by conjugating Wm to hyaluronic acid (HA), a biopolymer known for its transdermal delivery properties. In a bleomycin (BLM)-induced murine model of fibrosis, we assessed the therapeutic efficacy of the HA-Wm conjugate and its capacity to penetrate the skin barrier. Topical application of the HA-Wm conjugate exhibited enhanced skin permeation relative to free Wm, resulting in marked reductions in both dermal layer thickness and collagen accumulation within the fibrotic lesions. Mechanistically, HA-Wm treatment markedly decreased the numbers of α-smooth muscle actin-positive myofibroblasts and CD68-positive macrophages in the fibrotic skin. The HA-Wm treatment inhibited macrophage migration in vitro and reduced the serum concentrations of interferon-γ and tumor necrosis factor-α in BLM-treated mice. Importantly, the therapeutic effects of HA-Wm were abolished in *Fpr2*-deficient mice, confirming an FPR2-dependent mechanism of action. Collectively, these results demonstrate that topical treatment of HA-Wm alleviates skin fibrosis and inflammation via an FPR2-dependent pathway, representing a promising noninvasive therapeutic avenue for fibrotic skin disorders such as systemic sclerosis.

## Introduction

The primary hallmark of systemic sclerosis (SSc) is the overwhelming accumulation of collagen in connective tissues, which causes widespread fibrosis and tissue stiffening across various organs [1]. The progression of SSc fibrosis is intrinsically linked to the phenotypic switch of quiescent fibroblasts into highly contractile myofibroblasts expressing α-smooth muscle actin (α-SMA). These myofibroblasts are principally responsible for overproduction of extracellular matrix (ECM) proteins, which contributes to pervasive tissue induration [2]. The formation of myofibroblasts from fibroblasts is largely promoted by various inflammatory mediators and pro-fibrotic cytokines. Indeed, fibrotic progression in SSc is considered to be driven primarily by the infiltration of inflammatory cells and the ensuing activation of pro-fibrotic cytokine signaling cascades [3]. Although existing anti-inflammatory agents, such as methotrexate, synthetic corticosteroids, and cyclophosphamide, have shown some therapeutic efficacy, their use is often limited by adverse effects and complications [4]. The clinical management of SSc continues to be a significant hurdle, notwithstanding the ongoing attempts to develop effective therapeutic approaches [5]. Therefore, there is a pressing demand for innovative therapeutics capable of safely and effectively mitigating the chronic inflammation associated with SSc.

Formyl peptide receptors (FPRs), a class of G protein-coupled receptors, serve as pivotal regulators of immune surveillance and inflammatory signaling pathways [6]. These receptors are broadly distributed among immune and stromal cells, including monocytes, neutrophils, macrophages, and fibroblasts [7]. In humans, 3 distinct FPR subtypes—FPR1, FPR2 (also referred to as ALX), and FPR3—have been identified, whereas mice possess 2 homologous receptors, Fpr1 and Fpr2, which are functionally analogous to human FPR1 and FPR2 [8]. Increasing evidence indicates that FPR2 participates in immune dysregulation and tissue fibrosis observed in the skin and liver [9,10]. The activation of FPR2 is triggered by multiple ligand classes, including lipid-derived mediators (lipoxins and resolvins) and synthetic peptides exemplified by WKYMVm (Wm) [11]. Wm has demonstrated dual efficacy in reducing inflammation and fibrotic markers through FPR2 activation within the bleomycin (BLM)-induced scleroderma mouse model [9], indicating its promising therapeutic utility as a novel treatment approach for SSc. Moreover, administration of Wm has also been shown to reduce hyperoxia-induced pulmonary damage in neonatal mice [12]. However, the clinical translation of Wm is significantly hampered by its sub-hour biological half-life (*t*_1/2_ < 1 h) following systemic administration [13] and a molecular weight constraint that prevents passive transdermal flux [14]. This necessitates reliance on invasive administration, such as subcutaneous (s.c.) injection, to achieve therapeutic concentrations in the fibrotic dermis. Furthermore, repeated invasive administration can cause skin injury [15], infection [16], pain during injection [17], and neuropathic pain [18], underscoring the urgent need for a noninvasive platform to achieve efficient Wm-based treatment of SSc.

Hyaluronic acid (HA) is a glycosaminoglycan found naturally within the dermal ECM, characterized by its distinctive viscoelasticity and moisture retention properties. Beyond its vital biological functions in tissue hydration, lubrication, and facilitating cell-to-cell communication, HA exhibits excellent biocompatible and low immunogenicity [19]. Crucially for topical administration, HA is capable of penetrating the stratum corneum, the fundamental structural barrier for the skin [20]. Its hygroscopic nature allows it to hydrate the stratum corneum, thereby increasing skin permeability [21]. Moreover, the HA backbone contains hydrophobic patch domains consisting of 8 CH groups [22], which further enhance its skin penetration capacity. These physicochemical properties position HA as an effective transdermal delivery system, as evidenced by the successful delivery of various therapeutic proteins and peptides, including growth hormones, erythropoietin, epidermal growth factors, and parathyroid hormones [23–26]. In this study, we specifically selected HA with a molecular weight of 100 to 130 kDa. This range was chosen to balance delivery efficiency with biological safety; HA within the 20 to 200 kDa window demonstrates markedly enhanced skin permeation efficiency compared to the higher molecular weight of HA (>500 kDa), while avoiding the pro-inflammatory risks associated with low-molecular-weight HA oligomers (<20 kDa). Consequently, this molecular weight supports key physiological processes, including wound healing and tissue regeneration, making it ideal for treating fibrotic skin [27–29].

While HA conjugation is an established strategy for the transdermal delivery of proteins or peptides, its potential to enhance the transdermal delivery of the Wm peptide has not been explored in an SSc disease model. In this study, we developed and characterized a novel HA-Wm conjugate and hypothesized that conjugating Wm to HA would significantly enhance its skin penetration and therapeutic efficacy against skin fibrosis and inflammation. To evaluate this hypothesis, we utilized a BLM-induced mouse model of skin fibrosis to assess the delivery efficiency of HA-Wm and its ability to ameliorate fibrosis and inflammation. Furthermore, *Fpr2*-deficient mice were employed to determine whether the therapeutic effects of HA-Wm are specifically mediated by FPR2.

## Materials and Methods

### Materials

BLM powders were sourced from Tokyo Chemical Industry (Tokyo, Japan). The synthetic peptides, WKYMVm (Wm) and its tetramethylrhodamine-labeled analogue WK^TAMRA^YMVm (Wm^TAMRA^), were custom-synthesized by AnyGen Co., Ltd. (Gwangju, Republic of Korea). Sodium hyaluronate (100 to 130 kDa; Cat# SNR-HA-100K) was provided by SNvia Co., Ltd. (Busan, Republic of Korea). Primary antibodies were sourced as follows: anti-α-SMA (Cat# ab5694; Cambridge, UK), anti-vimentin (Cat# MAB-2105SP; R&D Systems, Minneapolis, MN, USA), and anti-CD68 (Cat# MCA1957GA; AbD Serotec, Raleigh, NC, USA) antibodies. Antibodies for arginase I (Cat# PA5-29645) and phospho-SMAD3 (Cat# 44-246G) were sourced from Thermo Fisher Scientific (Waltham, MA, USA). The total collagen analysis kit was purchased from QuickZyme Biosciences (Leiden, Netherlands), and enzyme-linked immunosorbent assay (ELISA) kits for mouse tumor necrosis factor-α (TNF-α) (Cat# 430904) and mouse interferon-γ (IFN-γ) (Cat# 430804) were purchased from BioLegend (San Diego, CA, USA).

### Preparation and analysis of the HA-Wm conjugate

Aldehyde-modified HA (HA-aldehyde) was prepared via a ring-opening reaction as previously reported [25]. Briefly, HA (10 mg/ml) was completely dissolved in 10 ml of deionized water. To initiate the vicinal diol oxidation, sodium periodate (NaIO_4_) was added into the HA solution at a stoichiometric ratio of 1:1 (NaIO_4_ to HA repeat unit). The mixture was agitated in the dark for 3 h, and the reaction was subsequently quenched by adding excess ethylene glycol. The resulting solution was purified by dialysis against deionized water for 3 days with multiple daily water changes and freeze-dried for 3 days to obtain HA-aldehyde as a white powder. The degree of aldehyde modification was determined by ^1^H-NMR (nuclear magnetic resonance) (AVANCE NEO 500, Bruker, Germany). For this analysis, HA-aldehyde was reacted with ethyl carbazate and sodium cyanoborohydride (at a 5-fold molar excess relative to HA repeating units) in pH 5.5 sodium acetate buffer (5 mg/ml) for 24 h. The resulting product was purified by dialysis for 3 days and freeze-dried prior to NMR analysis. The HA-Wm conjugate was prepared via reductive amination based on a published protocol [30]. HA-aldehyde (utilizing material with a 10 mol% modification degree) was dissolved in 8.4 ml of sodium acetate buffer and reacted with Wm (0.2 μM). This step facilitates the formation of Schiff base linkages between HA-aldehyde moieties and the N-terminal amino groups of Wm. After the initial conjugation, ethyl carbazate was added to quench any remaining aldehyde groups and allowed to react for 24 h. Subsequently, sodium cyanoborohydride was added and allowed to react for 24 h at room temperature to reduce the imine and hydrazone bonds, forming stable C–N linkages. The HA-Wm conjugate was characterized by size exclusion chromatography using high-performance liquid chromatography (HPLC, Nexera lite, Shimadzu, Japan). The system was equipped with a parallel-type double plunger pump, an SPD-40 UV-VIS detector, an autosampler SIL-40 series plate changer, and a Superdex 30 increase 10/300 GL column (Cytiva, Sweden). The mobile phase consisted of 30% acetonitrile and 0.1% trifluoroacetic acid, with a flow rate of 1 ml/min. Conjugation was confirmed by monitoring detection signals at 210 nm for HA and 280 nm for Wm. The degree of conjugation efficiency was calculated by comparing the molar ratio of Wm incorporated into the HA-Wm conjugates with the total amount of Wm initially added during the reaction, utilizing the absorbance signal at 280 nm specific to the tyrosine and tryptophan residues in Wm.

### BLM-induced skin fibrosis animal model

All protocols for animal experimentation were approved by the Institutional Animal Care and Use Committee at Pusan National University (Approval No. PNU-2022-0183) and fully complied with the National Research Council’s ethical guidelines for the Care and Use of Laboratory Animals. C57BL/6J male mice (22 to 24 g, 6 weeks of age) were obtained from Koatech in Gyeonggi-do, Republic of Korea. *Fpr2* knockout (KO) mice were kindly provided by Prof. R. J. Flower (Queen Mary University of London, UK). All animals were housed in a room where temperature and humidity were strictly controlled and given ad libitum access to both food and water. A BLM-induced skin fibrosis model was established in C57BL/6J mice, and animals were randomized into experimental groups. All groups received daily s.c. injections at a 1-cm^2^ shaved area of the dorsal skin for 3 consecutive weeks using a 27-gauge needle. The normal control group received daily phosphate-buffered saline (PBS; 100 μl s.c.), while the fibrosis control group received daily BLM (1 mg/ml in PBS; 100 μl s.c.) for 6 weeks. Treatment groups received daily BLM injections identical to the fibrosis control group and were additionally administered a concurrent daily treatment (100 μl) of PBS containing HA (0.1 μM), Wm (1 μM), or the HA-Wm complex (0.1 μM, corresponding to 0.55 μM Wm equivalents) either topically or subcutaneously from day 21 to day 42. Wm was administered via s.c. injection at a volume of 100 μl into a defined 1-cm^2^ area. Conversely, HA and HA-Wm were applied topically to the epidermis of a 1-cm^2^ area; 100 μl of each formulation was dispensed and gently rubbed onto the skin to ensure uniform coverage. No occlusive dressing was applied to the topical treatment sites, and the applied substances were confirmed to be uniformly distributed without runoff. Tissue and blood samples were subsequently collected 24 h after the final administration application.

### Histological analysis

Following euthanasia, dorsal skin samples were harvested and prepared for histopathological assessment. Skin tissue samples were fixed in 4% paraformaldehyde overnight. Following fixation, tissues were processed for paraffin embedding using standard protocols. Tissue sections were prepared from the paraffin-embedded blocks using a microtome and stained with hematoxylin and eosin (H&E) for visualization and subsequent quantification of the dermal layer thickness. For quantitative evaluation, 5 random regions per section were visualized at 10× magnification using a Carl Zeiss Axio Scan.Z1 system. Dermal thickness was quantified from the H&E-stained sections using ImageJ software (NIH) and was defined as the distance from the epidermal–dermal junction to the dermal–subcutaneous fat junction. Collagen deposition was evaluated using Masson’s trichrome staining and quantified using ImageJ software. Briefly, a color deconvolution plugin was applied to separate the blue-stained collagen fibers. The collagen-positive area was then measured and expressed as a percentage of the total tissue area per field.

### Hydroxyproline assay

To quantify total collagen content, hydroxyproline analysis was performed on mouse skin specimens. The Total Collagen Assay Kit (QuickZyme Biosciences, Leiden, Netherlands) was used according to the manufacturer’s protocol. Briefly, samples were weighed for normalization and then hydrolyzed in 50 mg/ml hydrochloride.

### Immunofluorescence analysis

For immunofluorescence analysis, formalin-fixed, paraffin-embedded skin sections were probed with various primary antibodies. Myofibroblasts and activated fibroblasts were identified using antibodies against α-SMA, vimentin, and p-SMAD3. Endothelial cells were identified using anti-ILB4 and anti-CD31 antibodies. Macrophages were identified using anti-CD68 (pan-macrophage) and anti-Arginase-1 (M2 macrophage) antibodies. Following primary antibody incubation, sections were subsequently probed with the appropriate secondary antibodies (Alexa Fluor 488 goat anti-rat antibodies, Alexa Fluor 488 goat anti-rabbit, or Alexa Fluor 568 goat anti-rabbit). After washing, slides were coverslipped using Vectashield mounting medium with DAPI (4′,6-diamidino-2-phenylindole) for nuclear counterstaining. An Olympus FluoView FV1000 laser confocal microscope was used to capture all fluorescence images. For quantitative analysis, ImageJ software was employed to enumerate the numbers of CD68^+^ macrophages and myofibroblasts. This cell counting was performed by 2 independent, blinded observers who assessed 5 randomly selected microscopic fields across 3 serial sections per sample.

### Analysis of transdermal delivery

Transdermal penetration of HA-Wm was evaluated using an ex vivo porcine ear model. Fresh porcine ears were sourced from a local abattoir and rinsed with PBS. The postauricular skin was carefully excised from underlying connective tissue and cartilage, trimmed of hair, and cut into strips. Circular skin samples (15 mm diameter) were then prepared and positioned between the donor and receptor chambers of a Franz diffusion cell (PermeGear, Hellertown, PA; 25 mm cell with a 4.91-cm^2^ total area) with the stratum corneum facing the donor compartment. The effective diffusion area was set at 1.76 cm^2^. After equilibrating the receptor medium at 32 °C, 250 μl of PBS containing Wm^TAMRA^ or HA-Wm^TAMRA^ was applied to the donor chamber. The receptor solution was continuously stirred at 600 rpm for 6 h.

To visualize the penetration in a disease context, dorsal skin tissues were isolated from both normal mice and mice with BLM-induced SSc. Skin samples were tape-stripped and mounted onto Franz diffusion cells as described above. Wm^TAMRA^ or HA-Wm^TAMRA^ was applied to the donor chamber of Franz diffusion cells, and the tissues were incubated at 32 °C for 24 h. Following incubation, the skin tissues were collected, fixed in 4% paraformaldehyde for 24 h, and cryo-embedded in Tissue-Tek O.C.T. compound at −70 °C. Cryosections (10 μm thickness) were prepared and rinsed with 1× PBS to remove residual O.C.T. compound. The processed sections were then imaged using an LSM900 confocal microscope (Carl Zeiss AG, Oberkochen, Germany) to visualize peptide penetration.

### ELISA analysis

To measure pro-inflammatory cytokine release, Raw 264.7 macrophages were first plated (1×10^5^ cells/well, 24-well plates) in DMEM (Dulbecco’s Modified Eagle Medium) containing 10% fetal bovine serum and acclimatized for 24 h (37 °C, 5% CO₂). Following stabilization, cells were simultaneously challenged with lipopolysaccharide (LPS; 1 μg/ml) and varying concentrations of HA, Wm, or the HA-Wm conjugate. Supernatants were retrieved 12 h later, and cytokine levels were quantified using a BioLegend commercial ELISA kit per the manufacturer’s instructions. Absorbance (405 nm) was measured on a TECAN microplate reader (TECAN Life Sciences, Männedorf, Switzerland), and concentrations were interpolated from a standard curve.

### Cell migration analysis

Macrophage migration was probed using Transwell inserts (Corning Costar, 5-μm pore). Raw 264.7 macrophages (1 × 10^5^ cells) were loaded into the upper chamber, while the lower chamber was filled with 600 μl of DMEM with LPS plus HA, Wm, or HA-Wm as the chemoattractant. After a 3-h incubation (37 °C, 5% CO₂), nonmigrated cells were mechanically removed from the top of the membrane via cotton swabs. The membranes were then fixed (4% paraformaldehyde), and cells on the undersurface were stained with 1 μl/ml Hoechst 33342 (Thermo Fisher Scientific) and rinsed. Images were acquired on a Nikon epifluorescence microscope (20× objective), and the total number of migrated cells was tallied from at least 9 random fields per membrane.

### Statistical analysis

All data are expressed as the mean ± SD. For multiple group comparisons, statistical significance was determined using one-way analysis of variance followed by Tukey’s post-hoc test to correct multiple comparisons. For comparisons between 2 independent groups, the Student’s *t* test was performed. *P* values < 0.05 were considered statistically significant. All statistical analyses were performed using GraphPad Prism software.

## Results

### Synthesis of the HA-Wm conjugate

**Fig. 1.**
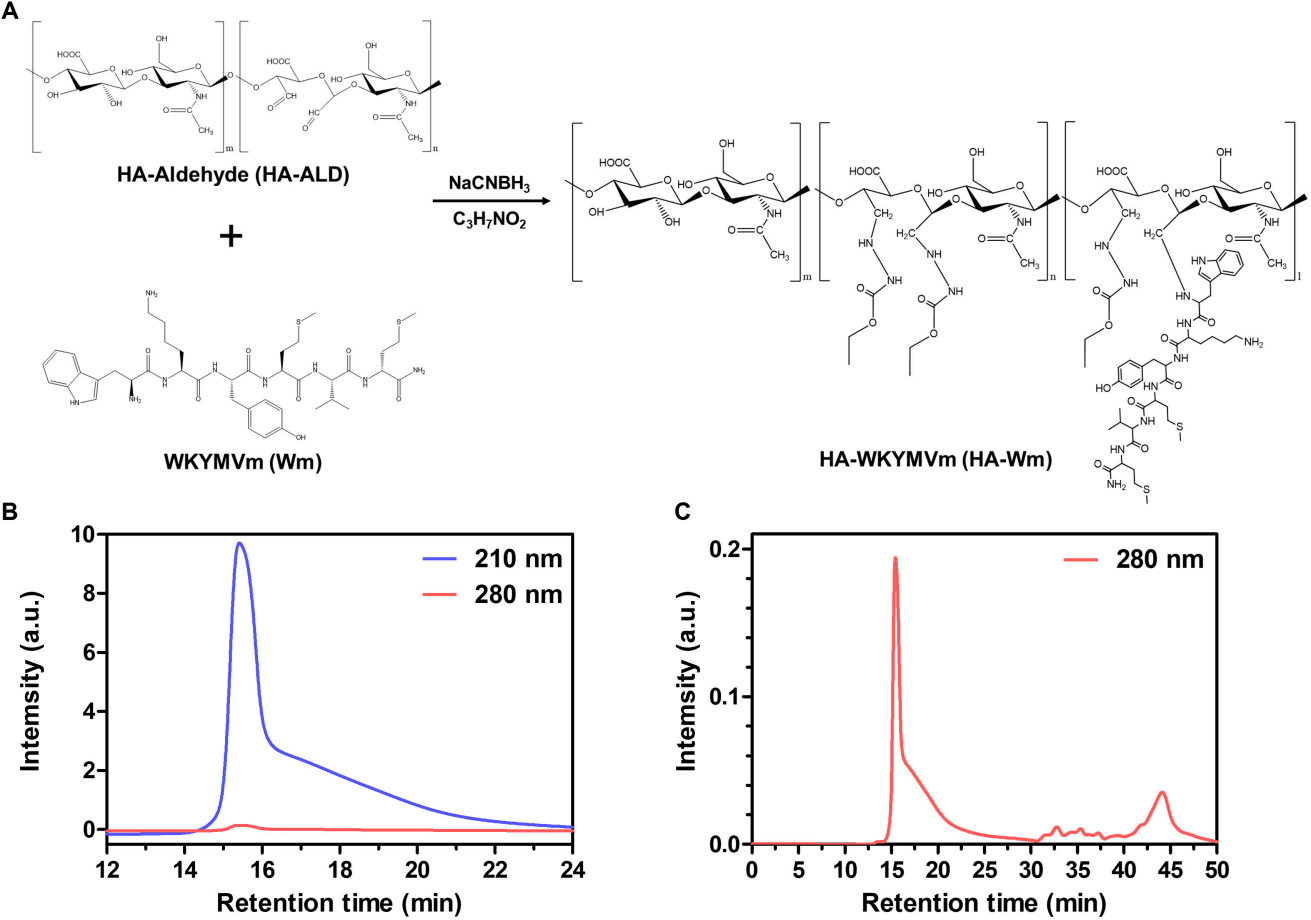
Synthesis and analysis of the HA-Wm peptide. (A) Schematic representation for the synthesis of HA-Wm. (B) High-performance liquid chromatogram (HPLC) of HA-Wm conjugate mixture detected at both 210 nm (blue) and 280 nm (red). (C) The HPLC profile showing only the 280-nm trace from (B). Arbitrary units (a.u.) of intensities are shown.

The HA-Wm conjugate was prepared via a site-specific coupling reaction as depicted in Fig. 1A. The reaction was conducted in pH 5.5 sodium acetate buffer, which leverages the p*K*_a_ difference between the N-terminal α-amino group and lysine ε-amino groups of Wm, thereby promoting selective conjugation to the HA-aldehyde moieties. Following the conjugation reaction, residual aldehyde groups were quenched using ethyl carbazate to prevent potential side effects from unreacted aldehydes. The successful formation of the HA-Wm conjugate was confirmed by HPLC (Fig. 1B and C) and NMR spectroscopy (Fig. S1). In the HPLC analysis, HA is primarily detected at 210 nm and the Wm peptide containing tyrosine and tryptophan is detected at 280 nm. The chromatogram showed a major peak at a retention time of approximately 15 min. This peak was detected at both 210 and 280 nm, which confirms the successful formation of the high-molecular-weight HA-Wm conjugate. In contrast, free, unconjugated Wm eluted significantly later at approximately 40 min, consistent with its much lower molecular weight. This free peptide peak was detected only at 280 nm, clearly distinguishing it from the conjugate. The degree of bioconjugation was determined by the molar ratio of Wm in the HA-Wm conjugates to the total Wm amount introduced initially for the conjugation. In this study, 9 Wm molecules were added per HA chain, resulting in a bioconjugation efficiency of 61%, which corresponds to approximately 5.5 Wm molecules conjugated to each HA chain.

### Transdermal delivery of the HA-Wm conjugate ex vivo

**Fig. 2.**
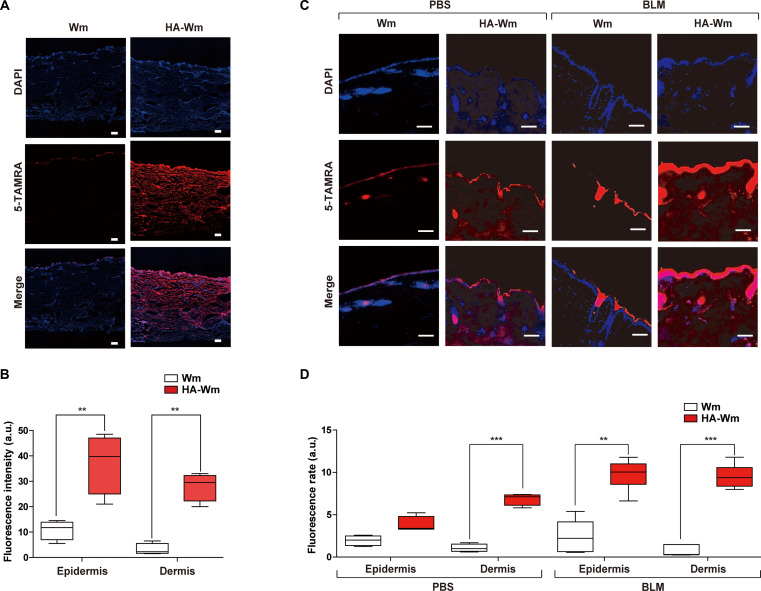
Transdermal delivery of HA-Wm ex vivo. (A) Representative confocal microscopy images of porcine ear skin 6 h after ex vivo topical administration of WmTAMRA or HA-WmTAMRA using a Franz diffusion cell. Scale bar = 100 μm (10× magnification). (B) Quantification of the mean fluorescence intensity of 5-TAMRA in the epidermal and dermal layers from the ex vivo experiment in panel (A); arbitrary fluorescence intensities (a.u.) of 5-TAMRA measured by confocal imaging are shown. (C) Representative confocal microscopy images of mouse dorsal skin after ex vivo topical administration of WmTAMRA or HA-WmTAMRA using a Franz diffusion cell. Images were taken from both healthy (PBS-treated) and BLM-induced fibrotic skin. Scale bar = 100 μm (20× magnification). (D) Quantification of the mean fluorescence intensity of 5-TAMRA in the epidermis and dermis area from the in vivo experiment in panel (C). Results are expressed as mean ± SD. **P < 0.01; ***P < 0.005 versus Wm (*n* = 6).

To validate the transdermal delivery of HA-Wm, we first conducted an ex vivo penetration assay using porcine ear skin mounted on a Franz diffusion cell apparatus. Porcine ear skin is a relevant model due to its morphological and permeability similarities to human skin [31]. Biopsy discs of porcine skin were positioned on the Franz diffusion cell, orienting the stratum corneum toward the donor compartment, while the receptor compartment was filled with PBS. Fluorescently labeled Wm (Wm^TAMRA^) or HA-Wm (HA-Wm^TAMRA^) was applied to the donor part. After 6 h, the skin tissues were collected, sectioned, and visualized via confocal microscopy. The fluorescence of HA-Wm^TAMRA^ was detected throughout the epidermis and deep into the dermis (Fig. 2A). In contrast, fluorescence from unconjugated Wm^TAMRA^ was minimal and confined to the superficial epidermis or stratum corneum. Quantitative analysis confirmed that the fluorescence intensity of HA-Wm^TAMRA^-treated skin was higher in both the epidermis and dermis compared to that of the Wm-treated skin (Fig. 2B). Furthermore, the transdermal delivery of HA-Wm^TAMRA^ occurred in a time-dependent manner (Fig. S2).

To test whether this enhanced delivery persists under pathological conditions, we compared ex vivo transdermal penetration of HA-Wm^TAMRA^ with Wm^TAMRA^ in both healthy and BLM-induced fibrotic mouse skin by Franz diffusion cell assay. Consistent with the permeability data obtained using porcine skin, topical administration of HA-Wm^TAMRA^ resulted in markedly greater fluorescence intensity within the dermis of healthy mice compared to unconjugated Wm^TAMRA^. Crucially, this superior penetration was also observed in fibrotic skin, where HA-Wm^TAMRA^ produced a robust fluorescent signal throughout both the epidermis and dermis (Fig. 2C). Intriguingly, a direct comparison revealed that the overall transdermal permeability of HA-Wm^TAMRA^ was even greater in fibrotic skin than in healthy, normal skin (Fig. 2D). These findings demonstrate that HA conjugation is a highly effective strategy for enhancing the transdermal delivery of the Wm peptide. More importantly, they show that its delivery efficiency is not hindered, but is in fact augmented, within the fibrotic tissue.

### Topical application of HA-Wm alleviates BLM-induced skin fibrosis

**Fig. 3.**
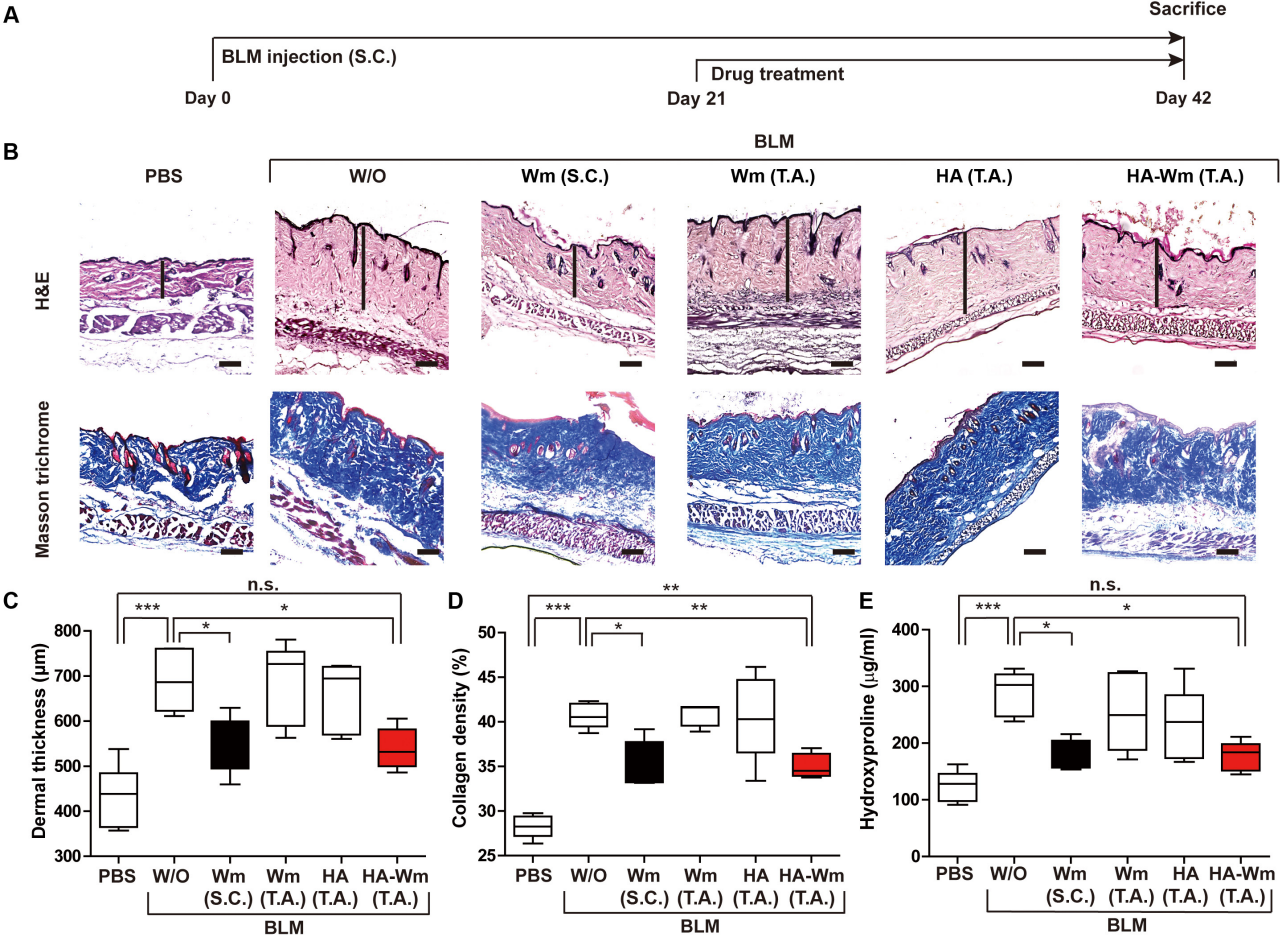
Effects of HA-Wm on BLM-induced skin fibrosis. (A) Schematic representation showing treatment time of BLM and drugs (Wm, HA, and HA-Wm). BLM was subcutaneously injected into the dorsal skin of mice for 6 weeks and the mice were treated daily with PBS containing Wm (1 μM), HA (0.1 μM), or HA-Wm (0.1 μM) from day 21 to day 42 via s.c. injection or topical administration. (B) H&E and Masson’s trichrome staining of skin (scale bar = 100 μm; 10× magnification). The black line indicates dermal thickness. (C) Quantification of dermal thickness from H&E staining. (D) Quantification of collagen density from Masson’s trichrome staining. (E) Effects of HA-Wm treatment on dermal hydroxyproline levels elevated by BLM. Results are presented as mean ± SD (*n* = 6 per group). *P < 0.05; **P < 0.01; ***P < 0.005; n.s., not significant.

To assess the in vivo therapeutic efficacy of HA-Wm, we utilized a murine skin fibrosis model. As shown in the schematic, mice received daily BLM via s.c. injections for 6 consecutive weeks. Treatment was administered daily from week 3 to week 6, concurrent with continued BLM injections (Fig. 3A). A preliminary dose–response study indicated that topical HA-Wm reduced fibrosis, with 0.1 μM HA-Wm markedly decreasing both dermal thickness and collagen content (Fig. S3). Therefore, we compared the efficacy of topical HA-Wm (0.1 μM, corresponding to 0.55 μM Wm equivalents) against key control experiments: s.c. Wm (1 μM), topical Wm (1 μM), and topical HA (0.1 μM). Histological analysis by H&E staining showed that mice in the BLM control group developed a notable increase in dermal thickness. This was markedly attenuated by both topical HA-Wm and s.c. Wm administration. In contrast, topical administration of HA or Wm had no significant effect (Fig. 3B and C).

To determine whether the topical administration of HA-Wm could reduce the increase in collagen in the fibrotic skin, we measured collagen levels using Masson’s trichrome staining. The BLM-induced increase in collagen density was markedly attenuated by topical HA-Wm and s.c. Wm, but was not affected by topical treatment of HA or Wm (Fig. 3B and D). To confirm these histological results, collagen levels were quantified by measuring the levels of hydroxyproline, a major component of collagen [32]. Hydroxyproline was increased in skin specimens from BLM-injected mice, and this increase was successfully attenuated by either topical HA-Wm or s.c. Wm treatment, but not by topical administration of HA or Wm (Fig. 3E).

### Topical treatment of HA-Wm reduces myofibroblast activation in BLM-induced skin fibrosis

**Fig. 4.**
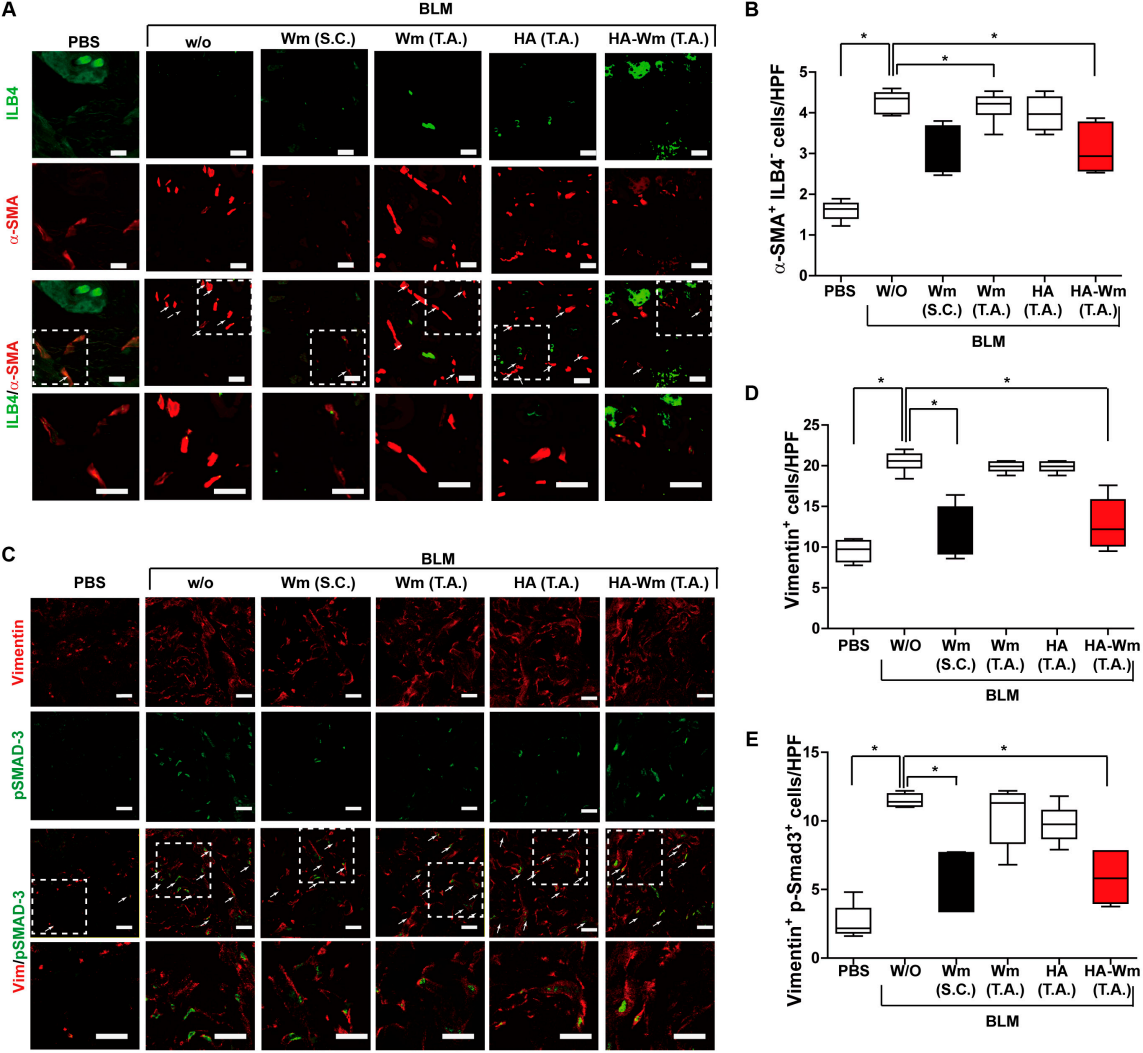
Effects of HA-Wm treatment on myofibroblast differentiation in vivo. (A) Representative immunofluorescence images of skin sections (from the groups in Fig. 3) costained for α-SMA (red) and the endothelial marker ILB4 (green). White arrows indicate α-SMA^+^ILB-4^−^ myofibroblasts. (B) Quantification of the number of α-SMA^+^ILB-4^−^ cells. (C) Representative immunofluorescence images of skin sections costained for vimentin and p-Smad3. Effects of HA-Wm on vimentin^+^ myofibroblast differentiation in BLM-injected skin. White arrows indicate vimentin^+^p-SMAD3^+^ cells. (D) Quantification of the total vimentin^+^ cells. (E) Quantification of the vimentin^+^/p-SMAD3^+^ cells. Results are expressed as mean ± SD (*n* = 6 per group). *P < 0.05. Images were captured by a confocal microscopy under a high-power field (40× magnification; scale bar = 50 μm). Regions within white dashed boxes are shown magnified in the panels below.

The transition of fibroblasts to myofibroblasts is a hallmark of SSc-associated tissue fibrosis [1]. To determine the anti-fibrotic effect of HA-Wm, immunostaining was conducted on the mouse skin samples. We identified myofibroblasts as cells expressing the myofibroblast marker α-SMA while lacking the endothelial cell marker ILB4. A marked accumulation of these α-SMA^+^/ILB4^−^ myofibroblasts was observed in BLM-administered skin. This BLM-induced increase was markedly diminished by topical treatment with HA-Wm or s.c. injection of Wm, but it was unaffected by topical administration of HA or Wm (Fig. 4A and B). These results were further supported by using CD31 as an alternative endothelial marker. The population of α-SMA^+^/CD31^−^ cells was likewise diminished following either topical administration of HA-Wm or s.c. injection of Wm (Fig. S4). Furthermore, an increase in vimentin-positive cells, another marker for myofibroblasts, was noted in BLM-exposed skin. This increase was also inhibited by topical administration of HA-Wm or the s.c. injection of Wm (Fig. 4C and D). Given that fibroblast activation is associated with heightened Smad3 phosphorylation [33]. We next quantified cells coexpressing vimentin and phosphorylated Smad3. BLM injection markedly increased the population of vimentin^+^/p-Smad3^+^ cells. Conversely, this elevation was markedly mitigated by either topical HA-Wm application or s.c. Wm injection. In contrast, topical treatment with HA or Wm did not affect this cell population (Fig. 4E). These results suggest that HA-Wm exerts its anti-fibrotic effect by inhibiting the accumulation and activation of myofibroblasts in fibrotic skin.

### HA-Wm exerts anti-inflammatory effects both in vitro and in vivo

To evaluate the anti-inflammatory properties of HA-Wm, we first performed in vitro assays using RAW 264.7 macrophages. Cells were stimulated with LPS in the presence of HA, Wm, or HA-Wm. The secretion of TNF-α was significantly heightened by LPS stimulation, and this effect was mitigated by cotreatment with either Wm or HA-Wm (Fig. S5A). Next, we assessed macrophage migration using a Transwell assay with LPS as the chemoattractant. LPS potently stimulated macrophage migration, and this LPS-induced chemotaxis was significantly inhibited by cotreatment with both Wm and HA-Wm (Fig. S5B). Importantly, none of the treatments (HA, Wm, or HA-Wm) impaired cell viability, regardless of the presence or absence of LPS (Fig. S5C). These in vitro data indicate that HA-Wm retains the anti-inflammatory functions of free Wm.

**Fig. 5.**
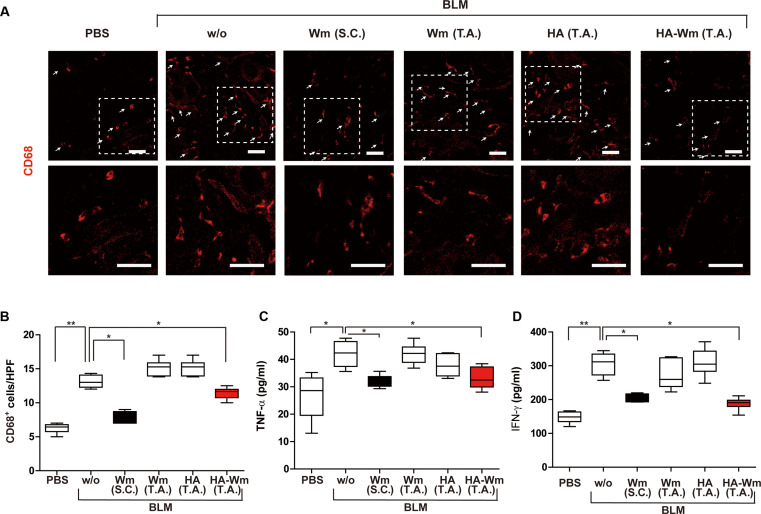
Effects of HA-Wm administration on macrophage infiltration and systemic inflammation in BLM-treated mice. (A) Representative immunofluorescence images of skin sections (from the groups in Fig. 3) stained with an anti-CD68 antibody (red color), and the CD68^+^ macrophages were indicated by white arrows. Regions within white dashed boxes regions are shown magnified below. Scale bar = 50 μm; 40× magnification. (B) Quantification of CD68^+^ cells per high-power field (HPF). (C and D) Effects of HA-Wm administration on systemic inflammatory cytokine levels in BLM-injected murine models. Systemic levels of TNF-α (C) and IFN-γ (D) were measured by ELISA. Results are presented as mean ± SD (*n* = 6 per group). *P < 0.05; **P < 0.01.

We next investigated the impact of topical HA-Wm on inflammatory cell infiltration in vivo. The elevated population of CD68^+^ macrophages in BLM-induced fibrotic skin was markedly diminished by either s.c. Wm injection or topical HA-Wm treatment (Fig. 5A and B). Furthermore, the number of Arginase-I-positive M2-type macrophages was also reduced by HA-Wm treatment (Fig. S6). Increased concentrations of inflammatory cytokines are frequently reported in the serum of individuals suffering from SSc [34]. In our preceding investigation, the s.c. administration of Wm attenuated the systemic concentrations of IFN-γ and TNF-α in BLM-treated mice [9]. Therefore, we assessed whether topical treatment of HA-Wm could affect systemic inflammation. Topical administration of HA-Wm significantly lowered the blood levels of IFN-γ and TNF-α in BLM-treated mice. However, the levels of IFN-γ and TNF-α in the blood of BLM-injected mice were not affected by topical treatment with Wm or HA. (Fig. 5C and D). These results suggested that topical treatment with HA-Wm ameliorates both local macrophage infiltration and systemic inflammation.

### The anti-fibrotic and anti-inflammatory efficacy of HA-Wm is dependent on FPR2

**Fig. 6.**
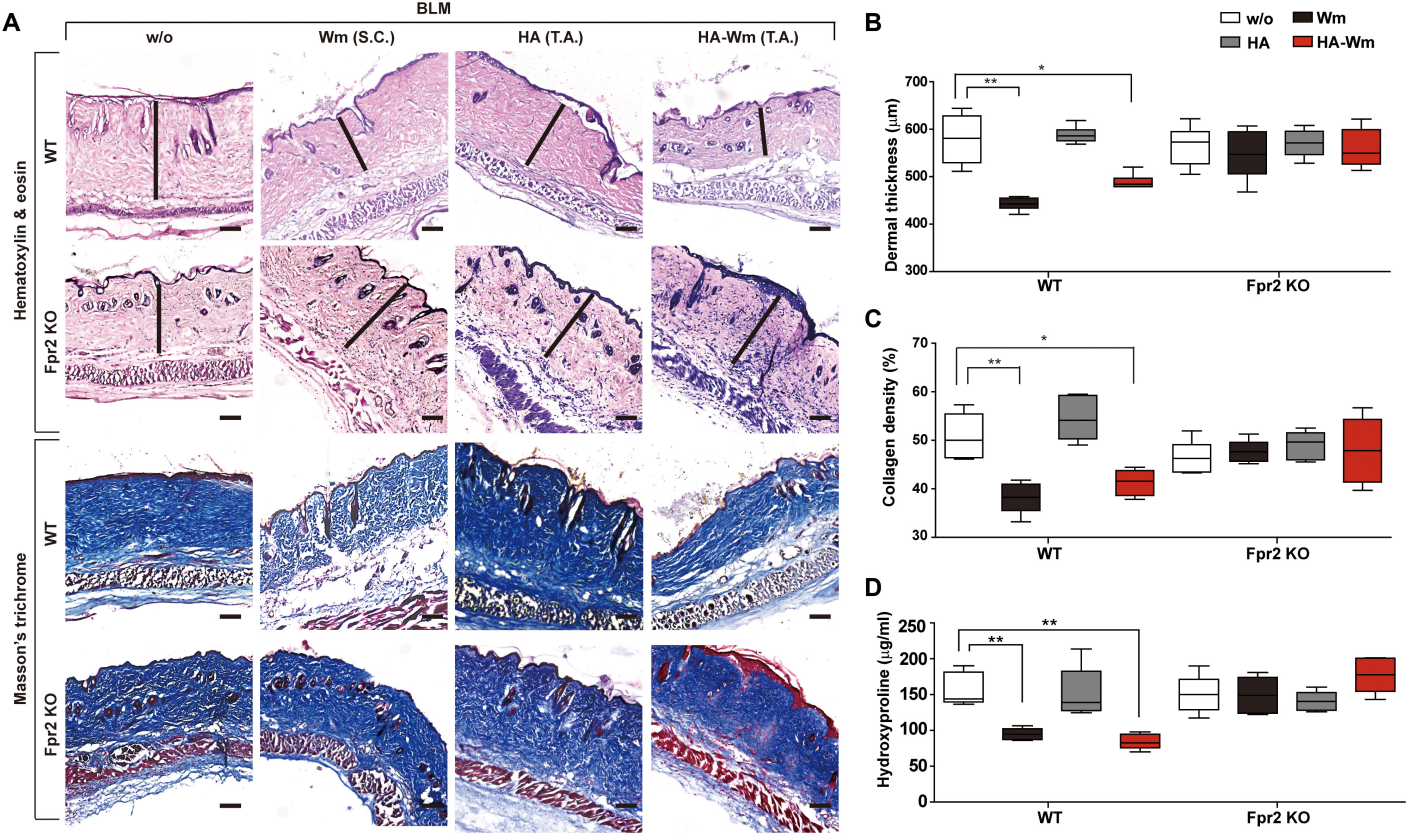
The anti-fibrotic effect of HA-Wm is FPR2-dependent. (A) Effects of HA-Wm on skin fibrosis in *Fpr2* KO mice. WT and Fpr2 KO mice were subjected to the 6-week BLM challenge, with or without topical HA-Wm (0.1 μM) treatment administered during the final 3 weeks (days 21 to 42), following the protocol described in Fig. 3A. Representative micrographs of skin sections after staining with H&E and Masson’s trichrome are shown. The black line indicates dermal thickness (scale bar = 100 μm; 10× magnification). (B) Measurement of dermal thickness from H&E-stained sections. (C) Measurement of collagen density from Masson’s trichrome-stained sections. (D) Quantification of total hydroxyproline concentrations in BLM-treated skin tissue. Results are presented as mean ± SD (*n* = 6 per group). *P < 0.05; **P < 0.01.

To determine whether the therapeutic activity of HA-Wm was dependent on FPR2, we compared its efficacy between *Fpr2* KO mice and wild-type (WT) controls. WT and *Fpr2* KO mice exhibited similar baseline skin thickness under normal conditions (Fig. S7A and B). Topical HA-Wm administration effectively attenuated skin fibrosis in WT animals; however, this anti-fibrotic activity was completely lost in *Fpr2* KO mice. Specifically, HA-Wm failed to ameliorate the BLM-induced increases in dermal thickness (Fig. 6A and B) and collagen fiber density (Fig. 6A and C) in *Fpr2* KO mice. Furthermore, the reduction in hydroxyproline concentration seen in WT animals was absent in *Fpr2* KO mice (Fig. 6D).

**Fig. 7.**
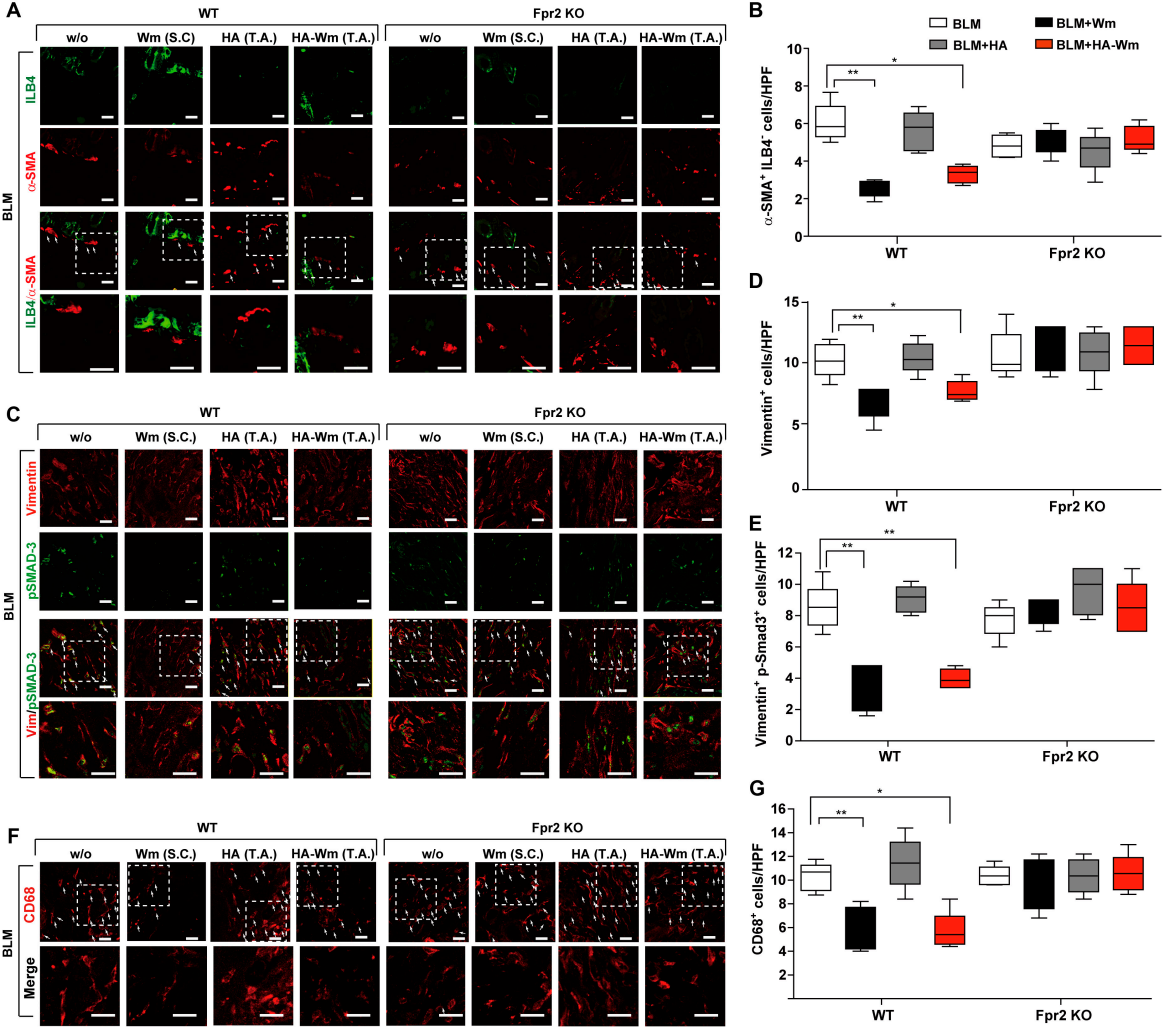
HA-Wm requires FPR2 to suppress myofibroblast and macrophage responses. (A) Representative immunofluorescence (IF) images of skin sections (from the groups in Fig. 6) costained for α-SMA (red) and ILB-4 (green). White arrows indicate α-SMA^+^/ILB-4^−^ myofibroblasts (B) Quantification of α-SMA^+^/ILB-4^−^ cells. (C) Representative immunofluorescence images costained for vimentin (red) and p-SMAD3 (green). White arrows indicate vimentin^+^/p-SMAD3^+^ cells. (D) Quantification of total vimentin^+^ cells. (E) Quantification of vimentin^+^/p-SMAD3^+^ cells. (F) Representative immunofluorescence images stained for CD68 (red). White arrows indicate CD68^+^ macrophages. (G) Quantification of CD68^+^ macrophages. Regions within white dashed boxes are shown magnified below. All images are 40× magnification; scale bar = 50 μm. Results are presented as mean ± SD (*n* = 6 per group). *P < 0.05; **P < 0.01.

We next investigated the involvement of FPR2 in HA-Wm-mediated inhibition of myofibroblast differentiation. Topical HA-Wm treatment caused a significant drop in the number of α-SMA^+^/ILB4^−^ myofibroblasts in the fibrotic skin of WT mice, but not in *Fpr2* KO mice (Fig. 7A and B). Similarly, HA-Wm administration decreased the number of Vimentin^+^ and Vimentin^+^/p-SMAD3^+^ myofibroblasts in WT mice; however, this mitigating effect was entirely absent in the *Fpr2* KO group (Fig. 7C to E). We subsequently investigated the involvement of FPR2 in modulating the inflammatory response. HA-Wm treatment significantly attenuated the infiltration of CD68^+^ macrophages in the fibrotic skin of WT mice, but this anti-inflammatory effect was not observed in *Fpr2* KO mice (Fig. 7F and G). Notably, the basal numbers of α-SMA^+^ myofibroblasts and CD68^+^ macrophages did not markedly differ between WT and *Fpr2* KO mice (Fig. S7C and D). Collectively, these data establish that FPR2 is required for HA-Wm to suppress myofibroblast differentiation and inflammation in the BLM model of dermal fibrosis.

## Discussion

Skin fibrosis, a hallmark of SSc, is driven by chronic inflammation and the subsequent pathological activation of fibroblasts [35–37]. Existing studies propose that the inflammatory cells promote the conversion of fibroblasts into myofibroblasts, the key players in the progression of fibrosis [36,38]. FPR2 is a critical mediator of immune resolution, the active process of re-establishing tissue homeostasis after inflammation [39]. Its ligands include not only the synthetic peptide Wm but also host-derived various ligands such as lipoxin and resolvin [40]. In the present study, we demonstrated that both free Wm and the HA-Wm conjugate effectively suppressed the LPS-induced chemotaxis of RAW 264.7 macrophages (Fig. S5B). Furthermore, topical treatment of HA-Wm diminished both the population of CD68^+^ macrophages in the BLM-injected skin and the systemic levels of IFN-γ and TNF-α in the BLM model. Crucially, the anti-fibrotic and anti-inflammatory effects of HA-Wm were completely abolished in *Fpr2* KO mice. This strongly positions HA-Wm as a novel, topically delivered therapeutic capable of in vivo FPR2 activation. Interestingly, the basal and BLM-induced systemic inflammation was comparable between WT and *Fpr2* KO mice (Fig. S8), suggesting that endogenous ligands alone are insufficient to activate this resolving pathway under fibrotic conditions, highlighting the need for an exogenous agonist like HA-Wm. While our in vivo analysis focused on macrophages and myofibroblasts, the therapeutic effects of HA-Wm may be broader. In addition to macrophages, FPR2 is also expressed in multiple immune and supporting cells, notably neutrophils, dendritic cells, and fibroblasts [6,7,41], suggesting that HA-Wm might also modulate these cell types to resolve inflammation.

A primary challenge for peptide therapeutics is effective delivery. Our strategy utilized HA, a biopolymer known for its skin penetration properties [22,42] and its role as a ligand for receptors like CD44, which is prevalent in the skin [43,44]. HA has been successfully used to deliver other macromolecules including growth hormones, epidermal growth factors, and parathyroid hormone peptides [23,24,42]. Furthermore, HA has been suggested as a drug delivery vehicle for inflammatory skin conditions, including atopic dermatitis and psoriasis [45]. Our ex vivo penetration studies validated this approach: HA-Wm^TAMRA^ successfully penetrated the epidermis and deep into the dermis, whereas free Wm^TAMRA^ was confined to the superficial epidermis (Fig. 2). This confirms that the HA-Wm conjugate functions as an efficient transcutaneous transport system that delivers the active Wm peptide to the target dermal tissue.

The clinical implications of this delivery system are significant. Subcutaneous injection of Wm inhibited fibroblast activation and inflammation in a skin fibrosis animal model [9]. Topical administration of HA-Wm (0.1 μM) achieved therapeutic effects comparable to a 10-fold higher concentration of s.c. injected Wm (1 μM) (Fig. 3). Based on our characterization, one HA-Wm molecule contains approximately 5.5 Wm peptides; thus, 0.1 μM HA-Wm corresponds to a theoretical Wm equivalent dose of 0.55 μM. Remarkably, this indicates that the topical conjugate achieved comparable therapeutic efficacy to the s.c. injection despite delivering approximately half the total peptide load (0.55 μM vs. 1 μM). This enhanced potency may be attributed to the superior skin retention properties of HA or the multivalent presentation of the peptide, although this estimate does not account for potential differences in bioavailability or receptor accessibility in vivo. This topical approach enables localized delivery, which could minimize systemic toxicity and enhance patient compliance by avoiding invasive injections. Intriguingly, this localized treatment still produced systemic anti-inflammatory effects. It is plausible that HA-Wm achieves this either via limited transdermal absorption into the circulation or, more likely, through local modulation of skin-resident immune cells that subsequently influence systemic immune tone. Further pharmacokinetic analyses are required to clarify this mechanism.

We also noted that HA-Wm permeability was higher in fibrotic skin than in healthy skin. This may be due to an impaired skin barrier, a condition often exacerbated by pruritus (itching) in SSc patients [46], suggesting that the therapeutic may be even more efficient where it is needed most. However, this finding must be validated in human scleroderma skin models. We acknowledge the limitations of the BLM-induced fibrosis model, which represents an acute inflammatory process rather than the chronic, progressive nature of SSc. Nonetheless, this model is highly relevant for studying the intersection of inflammation and fibroblast activation. Our data, showing that topical HA-Wm attenuated α-SMA and p-SMAD3 expression, consistently support the inhibition of this key fibrotic pathway. Given that SSc is a multiorgan disease [36], the application of BLM is known to provoke multiorgan fibrosis, with the most common manifestation occurring in the skin and the lungs [11]. However, BLM-induced fibrosis is an acute process and does not reflect the symptoms of progressive fibrosis in SSc. Therefore, it will be interesting to further investigate whether the topical administration of HA-Wm has a therapeutic effect on fibrosis of multiple organs in other disease models that mimic the pathology of SSc.

In conclusion, we report that HA conjugation is a highly effective strategy for the transdermal delivery of the Wm peptide. Topical HA-Wm alleviated BLM-induced skin fibrosis by inhibiting myofibroblast differentiation and inflammation. This therapeutic efficacy was unequivocally dependent on FPR2. Compared to conventional drugs often associated with severe side effects, the immune-resolving activity of HA-Wm offers a promising, safe, and effective therapeutic avenue for fibrotic skin disorders like SSc and potentially other related immunological diseases.

## Ethical Approval

The entirety of the animal experimentation adhered to protocols that received approval from the Institutional Animal Care and Use Committee at Pusan National University.

## Data Availability

All datasets that substantiate the conclusions of this article can be obtained from the corresponding authors on request.
