# Supplementary material for: Transdermal Delivery of Hyaluronate-Conjugated Formyl Peptide Receptor 2 Agonistic Peptide Ameliorates Bleomycin-Induced Skin Fibrosis
Source: Biomater Res. 2026 Mar 12;30:0316. doi: 10.34133/bmr.0316 (PMC12981301; doi:10.34133/bmr.0316)
Supplement: Supplementary 1 — Figs. S1 to S8 [file bmr.0316.f1.pdf]

Supplementary Information

Transdermal Delivery of Hyaluronate-Conjugated Formyl Peptide Receptor 2 Agonistic Peptide Ameliorates Bleomycin-Induced Skin Fibrosis

Gyu Tae Park[1¶], Hye Eun Choi[2¶], Jae-Kyung Lim[1¶], Eun-Bae Choi[1], Jeong-Hyun Park[1], Su Bin Lee[2], Moon-Bum Kim[3], Ki Su Kim[2*], and Jae Ho Kim[1*]

[1]Department of Physiology, College of Medicine, Pusan National University, Yangsan 50612, Gyeongsangnam-do, Republic of Korea; [2]School of Chemical Engineering, College of Engineering, Pusan National University, Busan 46241, Republic of Korea; [3]Department of Dermatology, Pusan National University Hospital, Busan 49241, Republic of Korea

*Address correspondence to: Jae Ho Kim, Ph.D. (jhkimst@pusan.ac.kr) and Ki Su Kim, Ph.D., (kisukim@pusan.ac.kr)

¶ These authors contributed equally to this work

Supplementary Materials and Methods

Cell survival assay

Cells were seeded in 96-well culture plates at a density of 1×10^4 cells/well and cultured for 24 hours prior to treatment. Cells were dose-dependently treated with HA, Wm, and HA-Wm in serum-free DMEM medium containing 1 μg/mL LPS for 12 hours, and then incubated with 10 μL EZ-CYTOX (Dogenbio, Seoul, Republic of Korea) for 2 hours at 37°C. After shaking the plates for 1 min, the absorbance of the solution was measured at 450 nm using an Infinity microplate spectrophotometer (Tekan Trading AG, Switzerland) and expressed as a relative percentage of the control

Supplementary Figures

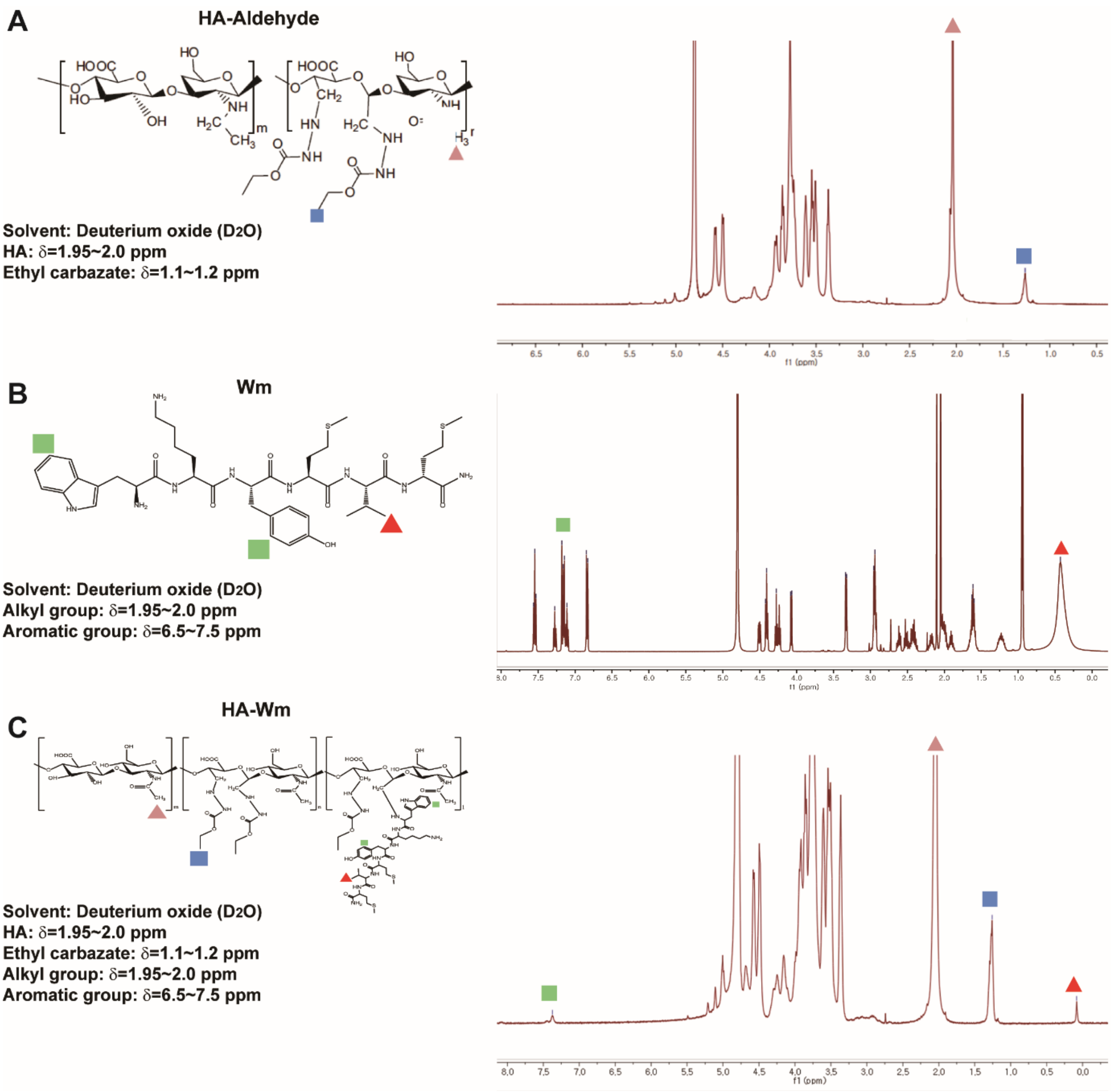

Figure S1. ^{1}H-nuclear magnetic resonance spectra of HA-aldehyde (A), Wm (B), and HA-Wm (C). The functional moieties (HA, ethyl carbazate, alkyl, aromatic groups) and their chemical shift values (δ, ppm) were indicated.

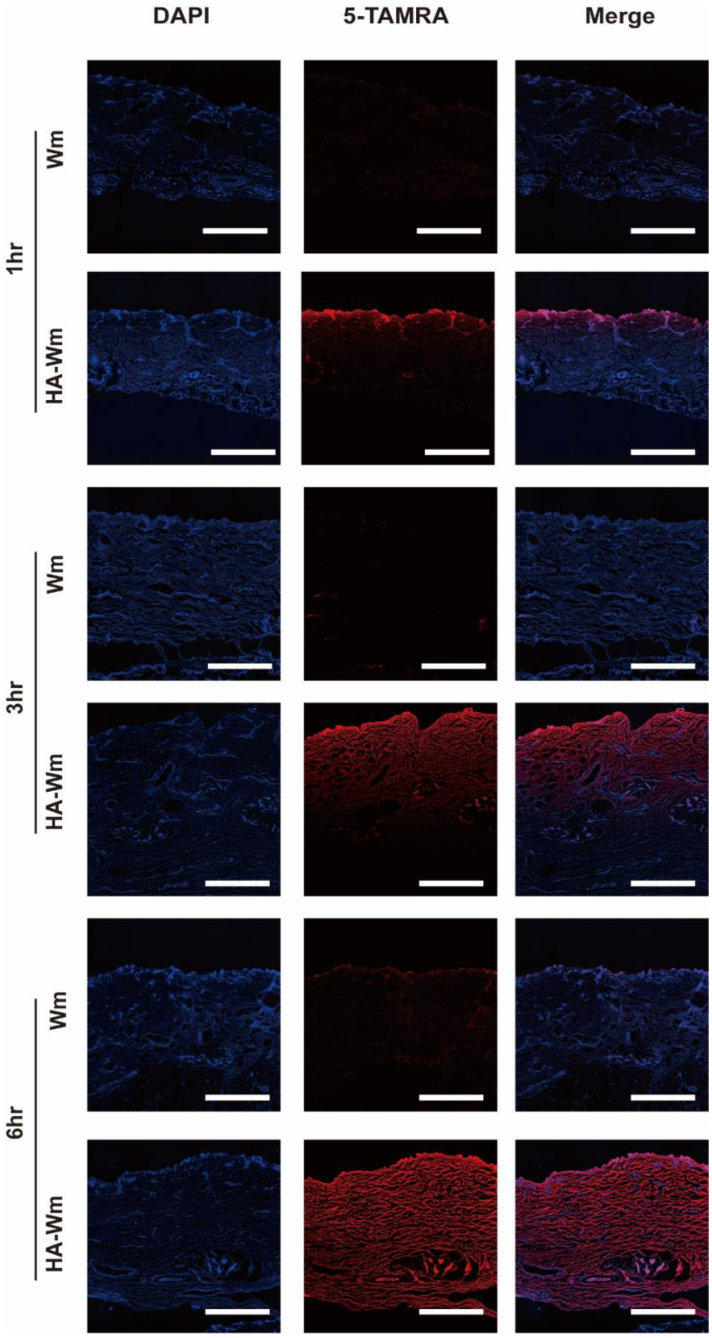

Figure S2. Time-dependent penetration of HA-Wm in porcine ear skin. Porcine ear skin samples were topically treated with Wm ^{TAMRA} and HA-Wm ^{TAMRA} and incubated using Franz diffusion cells for 1, 3, and 6 hours. Representative fluorescence images are each time point are shown. Scale bar = 1 mm.

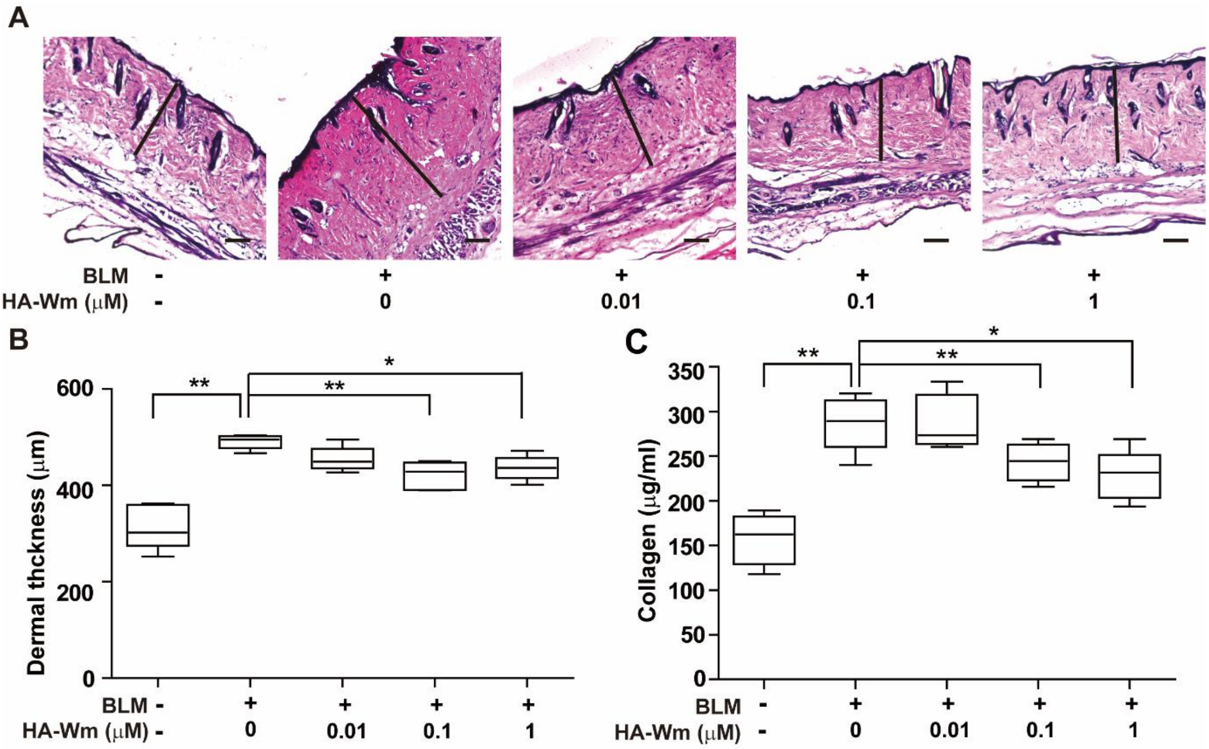

Figure S3. Dose-dependent effects of HA-Wm treatment on tissue fibrosis in a murine skin fibrosis model. (A) The BLM-induced skin fibrosis mice were topically treated with the increasing dose of HA-Wm daily for 3 weeks, and skin sections were stained using H&E and Masson's Trichrome staining kits. The dermal layer between the epidermal-dermal junction and the dermal-fat junction was indicated with an arrow on H&E-stained sections. Scale bar = 100 μm. (B) Dermal thickness was quantified from the H&E images. (C) Effect of HA-Wm treatment on the BLM-induced collagen accumulation in skin. Collagen content of the skin specimens was quantified as described in Materials and Methods section. Data represents mean ± SD (n = 8 per groups). *p < 0.05, ***p < 0.005.

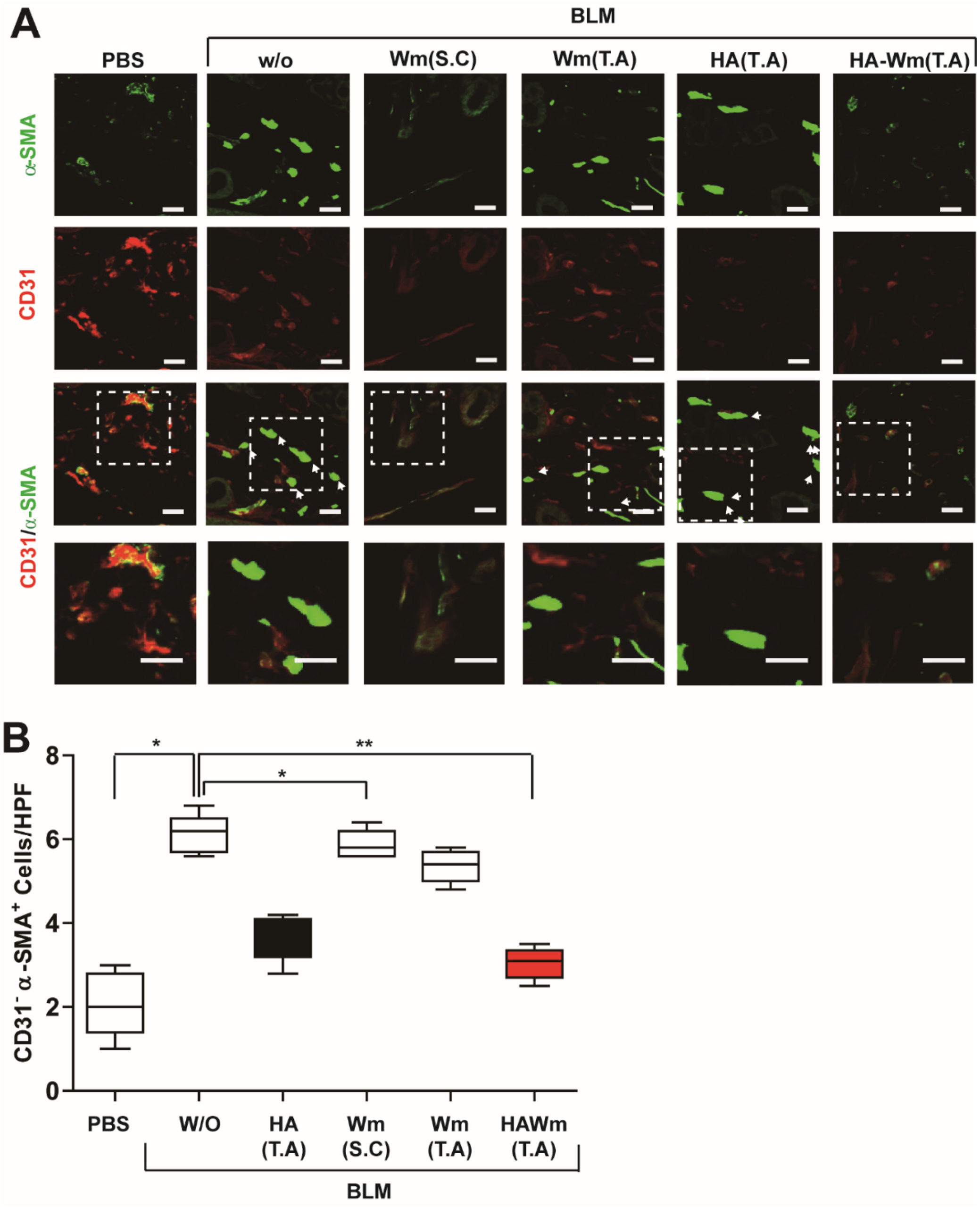

Figure S4. Effects of HA-Wm treatment on CD31-negative myofibroblast activation in BLM-induced skin fibrosis. (A) Effects of HA-Wm on α-SMA$^+$CD31$^-$ myofibroblast differentiation in BLM-induced skin fibrosis. Skin sections were stained with antibodies against α-SMA (green color) and CD31 (red color), and overlaid images are shown. Images were captured by a confocal microscopy under a high-power field (40× magnification; scale bar = 50 μm). The white dashed line box regions are shown at a magnified scale in the images below. (B) Quantification of α-SMA$^+$CD31$^-$ myofibroblasts under a high-power field. Data represents mean ± SD (n = 6 per group.) * $p < 0.05$, ** $p < 0.01$.

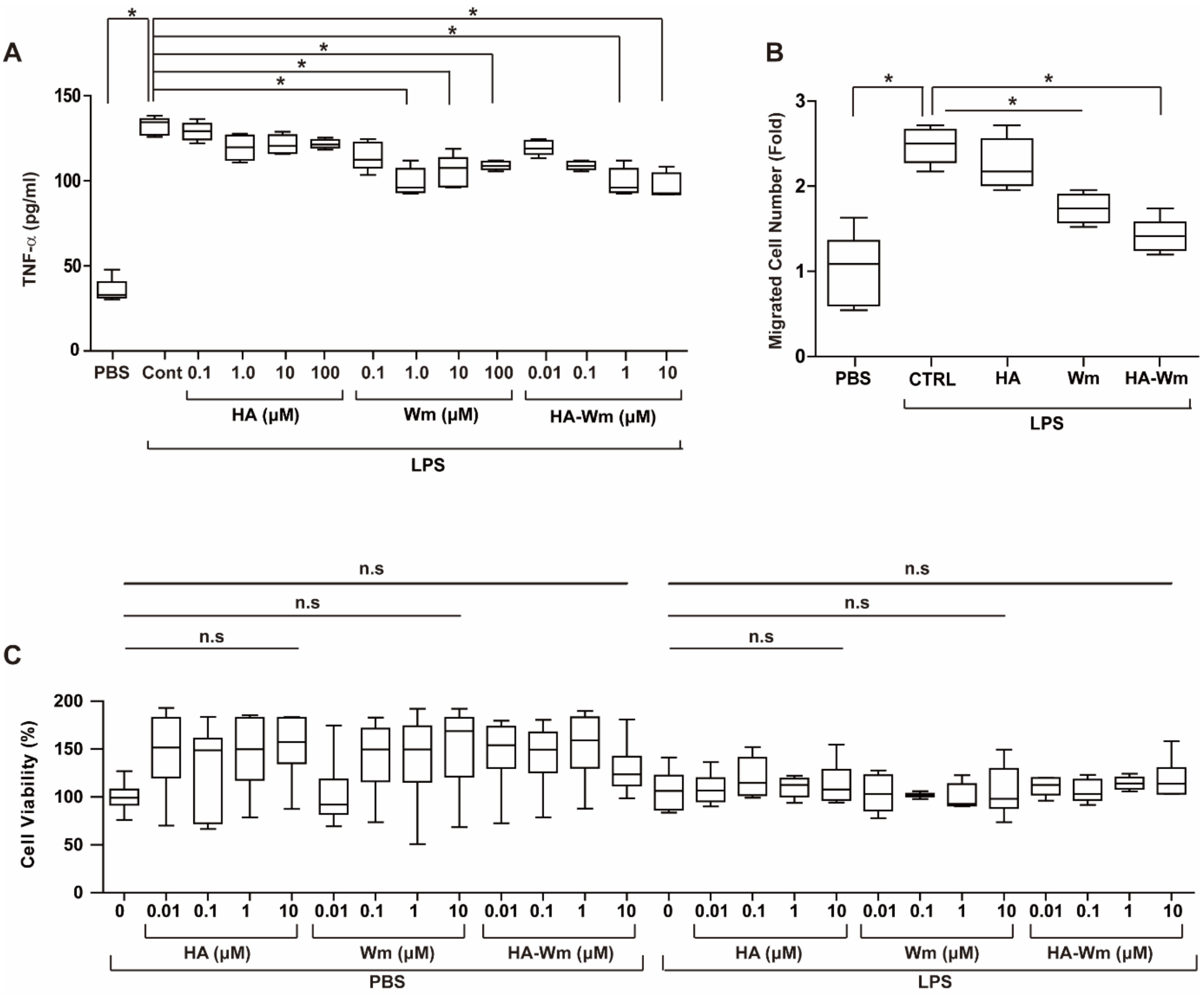

Figure S5. Effects of HA-Wm treatment on LPS-induced inflammatory activation of murine macrophages. (A) RAW 264.7 cells were treated with LPS (1 μg/mL) and increasing concentrations of HA, Wm, or HA-Wm for 12 h. TNF-α secretion from RAW 264.7 cells were measured via ELISA. (B) Migration of RAW 264.7 macrophages measured using chemotaxis chambers after treatment with LPS (1 μg/mL) and HA (0.1 μM), Wm (1 μM), or HA-Wm (0.1 μM) for 12 h. (C) Cell viability assessed in RAW 264.7 cells treated with the indicated concentrations of Wm, HA, or HA-Wm in the absence (PBS) of presence of 1 μg/mL LPS. Data represent mean ± SD (n = 6 per group). **p* < 0.05, n.s: non-significant.

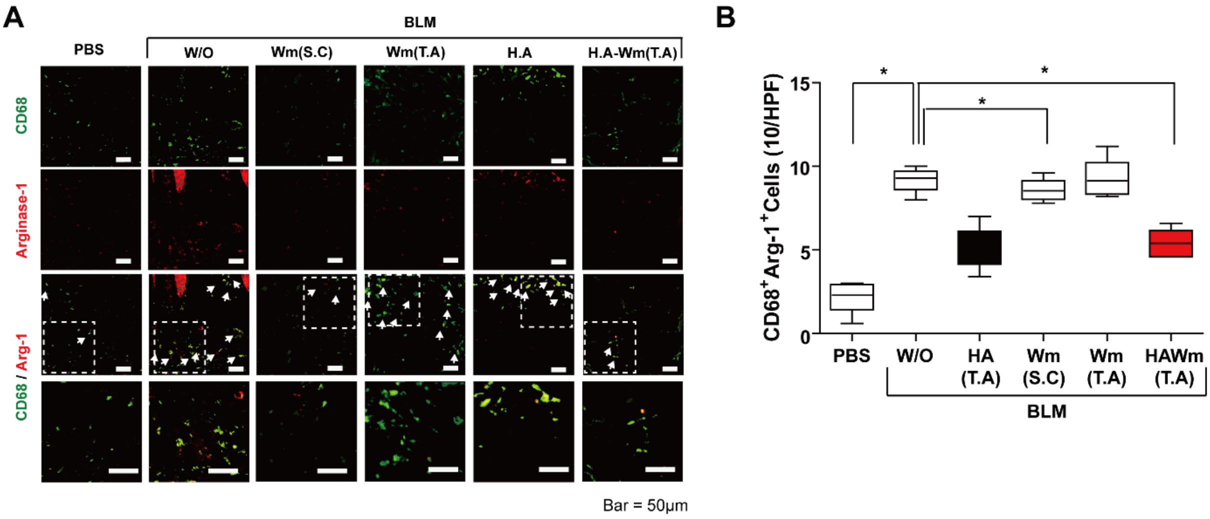

Figure S6. Effects of HA-Wm on the number of M2 macrophage in BLM-induced skin fibrosis. (A) Skin sections of BLM-injected mice (as shown in Fig. 3) were immunostained with antibodies against CD68 (green color) and Arginase-1 (a M2 macrophage marker; red color), and overlaid images are shown. Confocal microscopic images were acquired at 40× magnification; scale bar = 50 µm. The white dashed line box regions are shown at a magnified scale in the images below. (B) Quantification of CD68+Arginase-1-positive M2 macrophages per high-power field. Data represent mean ± SD (n = 6 per group). **p* < 0.05.

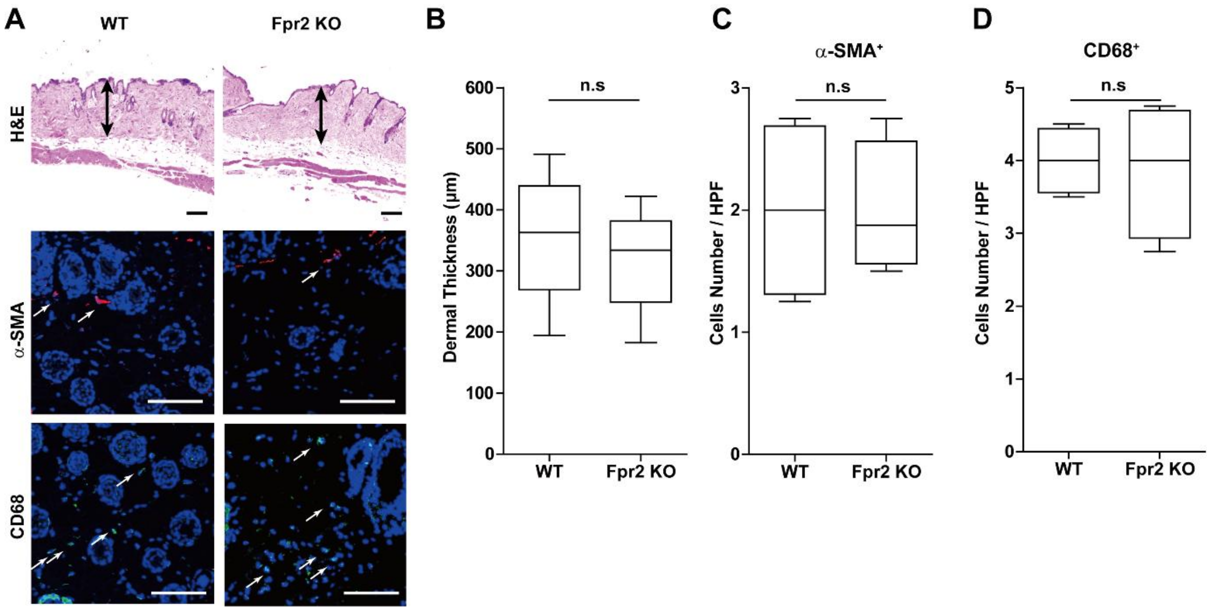

Figure S7. Basal levels of myofibroblasts and macrophages in the skin of WT and *Fpr2* KO mice. WT and *Fpr2* KO mice was subcutaneously injected with PBS daily for 42 days. Dorsal skin tissues were subjected to H&E staining and the black line indicates dermal thickness. Scale bar = 100 µm; 10× magnification. The skin specimens were stained with anti-α-SMA (red color) and anti-CD68 antibodies (green color), respectively. Nuclei were counterstained with DAPI (blue color), and overlaid images are shown. The α-SMA^{+} and CD68^{+} cells were indicated by white arrows. Scale bar = 50 µm. Dermal thickness (B), alpha SMA-positive myofibroblasts (C), and CD68-positive macrophages (D) were determined from the images. Data represent mean ± SD (n = 6 per group). n.s: non-significant.

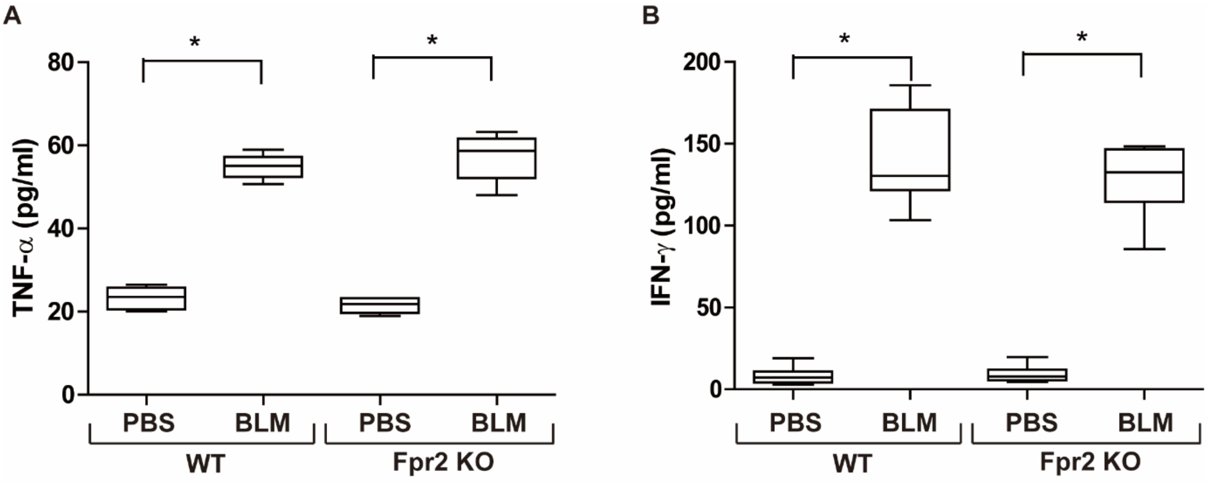

Figure S8. Comparison of inflammatory cytokine levels in WT and *Fpr2* KO mice with or without BLM administration. Wild-type WT and *Fpr2* KO mice were treated with vehicles or bleomycin (BLM), and serum levels of tumor necrosis factor-α (TNF-α; A) and interferon-γ (IFN-γ; B) were measured. Both basal (PBS) and BLM-induced cytokine levels were compared between WT and *Fpr2* KO groups. Data represent mean ± SD (n = 6 per group). *$p < 0.05$.
